# Persistence, Seasonal Dynamics, and Regional Differentiation of the Parasitic Mite *Tropilaelaps mercedesae* in *Apis mellifera* L. Colonies Under Non-Endemic Climatic Conditions

**DOI:** 10.3390/insects17090964

**Published:** 2026-09-18

**Authors:** Anna Z. Brandorf, Olga Frunze, Anna Bakulina, Ekaterina Bessolitsyna, Ulzhan Nuraliyeva, Merey Torekhanov, Oleg Krupskiy, Sultan Saurov, Hyung-Wook Kwon

**Affiliations:** 1 Rudnitsky Federal Agricultural Research Center of the North-East, Kirov 610007, Russia; 2 Department of Life Sciences & Convergence Research Center for Insect Vectors (CRCIV), Incheon National University, R&D Complex, Incheon 22012, Republic of Korea; frunzeon@gmail.com; 3 Limited Liability Partnership Kazakh Research Institute of Livestock and Fodder Production, Almaty 050035, Kazakhstan; 4 Group of Educational Programs “Crop production”, Kazakh Agro Technical Research University, Astana 010011, Kazakhstan

**Keywords:** biological invasion, brood infestation, honey bee health, seasonal dynamics, deformed wing virus, parasite ecology

## Abstract

*Tropilaelaps mercedesae* is an emerging parasite of *Apis mellifera* L. that is expanding beyond its traditional tropical range, yet its ecology in temperate regions remains poorly understood. This study investigated the establishment, seasonal infestation dynamics, morphometric variation, and virus carriage of *T. mercedesae* populations in three climatically contrasting regions of temperate Eurasia: Sochi and Makhachkala (Russia) and Zhetisay (Kazakhstan). Honey bee colonies were monitored throughout the annual cycle to assess infestation in relation to brood availability and seasonal climatic conditions. Morphometric variation was evaluated using six diagnostic body measurements, and virus carriage was determined by reverse-transcription polymerase chain reaction for seven major honey bee viruses. Infestation patterns of *T. mercedesae* differed among regions but consistently followed seasonal brood availability. Morphometric analysis revealed geographic differentiation among mite populations, with canonical discriminant analysis correctly classifying 70.9% of specimens. Four viruses were detected in the mites, with deformed wing virus present in all regions and the greatest virus diversity observed in Makhachkala. Together, these results indicate that *T. mercedesae* can establish persistent populations across a broader climatic range than previously recognized. They also provide important information for surveillance, risk assessment, and the development of management strategies for this emerging honey bee parasite.

## Simple Summary

Honey bees are essential for pollinating crops and wild plants, but their health is increasingly threatened by parasites and diseases. One of the most concerning emerging pests is the parasitic mite *Tropilaelaps mercedesae*, which has recently spread beyond its traditional tropical range into cooler parts of Eurasia. To better understand this expansion, we studied mite populations in honey bee colonies from southern Russia and southeastern Kazakhstan, where climate conditions differ considerably. We found that the mite was present every year in all study regions, showing that it can survive and establish stable populations outside its original range. Although infestation levels changed with the seasons, they closely followed the availability of developing bee brood, where the mites reproduce. We also observed regional differences in the mites’ physical characteristics and in the viruses associated with them. These findings improve our understanding of how *T. mercedesae* adapts to new environments and spreads into temperate regions. This information will help support earlier detection, more effective monitoring, and better management strategies to protect honey bee colonies and the pollination services they provide.

## 1. Introduction

The emergence of new parasites in managed honey bee colonies may be facilitated by the movement of *Apis mellifera* L. for honey production, crop pollination, and breeding, which can bring this species into contact with parasites historically associated with other species of the genus *Apis*. Successful host shifts can result in the establishment and subsequent geographic spread of new parasite–host associations.

A well-known example is *Varroa destructor*, which shifted from *Apis cerana* to *A. mellifera* L. during the twentieth century [1,2]. Following host switching, *V. destructor* spread rapidly throughout the world and is now regarded as the most economically important parasite of managed honey bees [3]. This example illustrates the potential consequences of successful host adaptation combined with the extensive movement of managed honey bees.

A comparable host shift has occurred in *Tropilaelaps mercedesae*. Historically associated with the giant Asian honey bee *Apis dorsata*, *T. mercedesae* subsequently shifted to the introduced western honey bee *A. mellifera* and became an important parasite of this host across much of Asia [4,5,6,7,8,9,10]. Unlike *V. destructor*, *T. mercedesae* is strongly dependent on developing brood for feeding and reproduction and cannot feed on adult honey bees; consequently, its dispersal phase on adults is brief [6]. Nevertheless, mites can be carried on adult bees leaving infested colonies, providing a potential mechanism for dispersal between colonies [11]. Infestation of developing bees can reduce emergence weight and adult longevity and promote deformed wing virus infection and associated clinical symptoms [5]. Severe infestation can result in brood damage, colony weakening and, if uncontrolled, colony mortality, with potentially substantial consequences for beekeeping [6]. However, comprehensive quantitative estimates of the economic losses specifically attributable to *T. mercedesae* remain limited.

**Figure 1:**
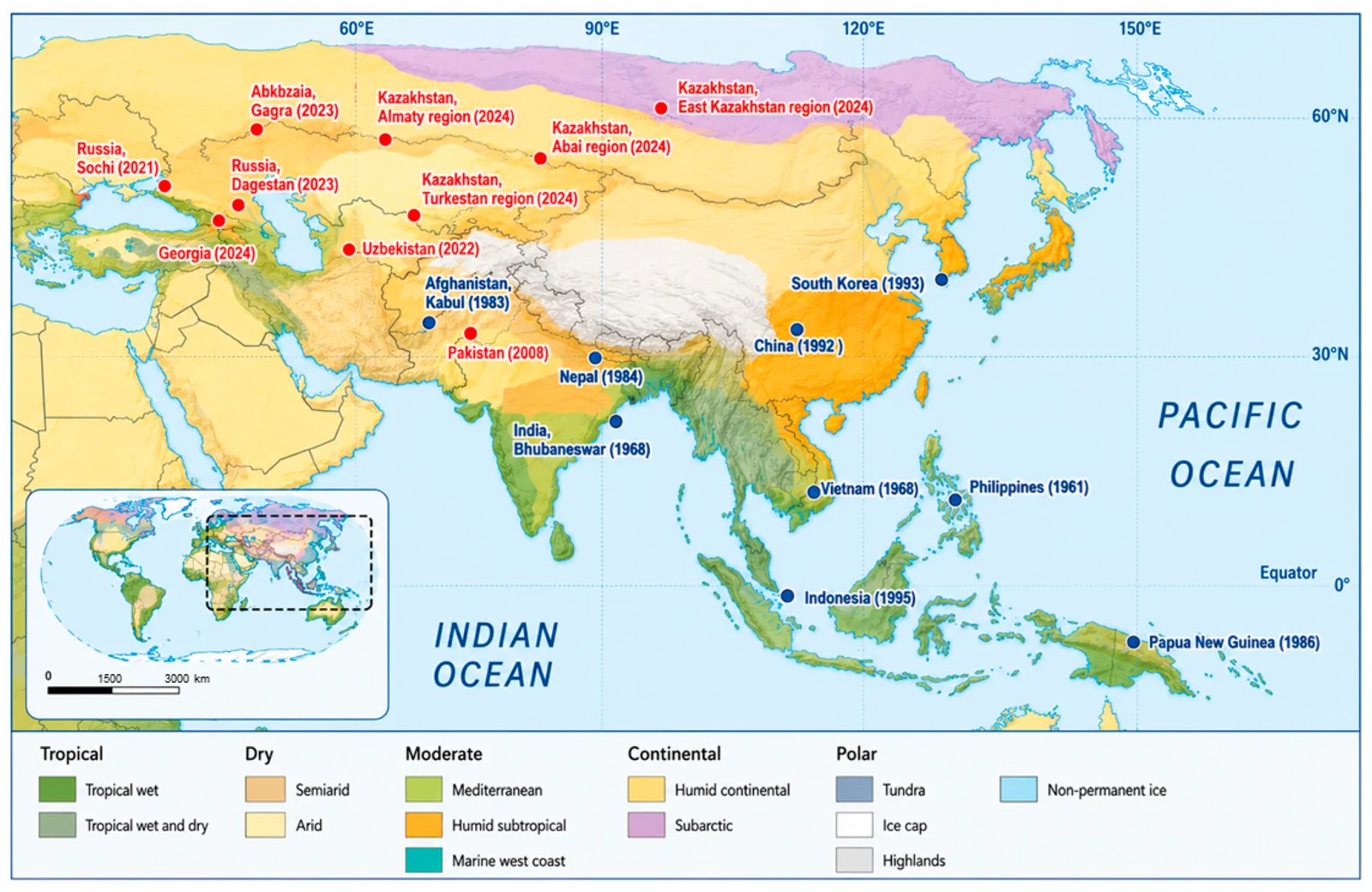
Reported geographic distribution of *T. mercedesae* in *A. mellifera* L. colonies across different climatic zones [2,4,7,8,9,10,13,16,17,18,20,21,22,23,24,25,26,27]. Countries or regions are labeled with the year of the first published record of *T. mercedesae* in *A. mellifera* L. Data sources are provided in Supplementary Table S1. Blue circles indicate records published before 2000, whereas red circles indicate records published in or after 2000.

This strong dependence on developing brood has been considered an important constraint on the geographic distribution of *T. mercedesae*. For many years, it was assumed that the mite would be unable to maintain stable populations in temperate regions where honey bee colonies experience prolonged broodless periods during winter [12]. Recent expansion of its known geographic range into Eurasia, however, increasingly challenges this assumption [13,14,15]. The first records of *T. mercedesae* in the Krasnodar Region (Russia) were reported in 2021, followed by the Republic of Dagestan (Russia) and Uzbekistan in 2022 [16,17], Georgia in 2024 [18], and the Republic of Kazakhstan in 2024 [19]. These records demonstrate the presence of the parasite in regions where honey bee colonies experience marked seasonal changes in brood availability. Together, these observations indicate that the known distribution of *T. mercedesae* has extended beyond its historically recognized tropical and subtropical range into regions with more strongly seasonal climates (Figure 1).

The increasing number of detections coincides with intensive international movement of honey bee packages. During the last 15 years, large numbers of bee packages have been imported annually from Central Asian countries into Russia. According to official data from the Russian veterinary information system “VETIS” (Ministry of Agriculture of the Russian Federation, 25 December 2025, No. 7-ΦC-A/3518-3226), more than 62,000 bee packages were imported in 2021, 115,000 in 2022, more than 31,000 in 2023, more than 88,000 in 2024 and more than 113,000 in 2025. Bee packages destined for Russia are transported through the Republic of Kazakhstan, although official data on imports into Kazakhstan are unavailable. The temporal coincidence between parasite detections and large-scale movement of managed bees identifies bee trade as a plausible dispersal pathway but does not establish a causal link. The precise route and geographic origin of *T. mercedesae* introduction into Russia and Kazakhstan therefore remain unknown.

One example suggesting that the movement of bee packages may facilitate the spread of *T. mercedesae* was reported by Joharchi and Stolbova [17]. During a survey in the Tyumen Region (Russia), *T. mercedesae* was detected in hive debris from colonies that had died during autumn at an apiary that had imported bee packages from Central Asia in the spring of 2023. Furthermore, in June 2026, *T. mercedesae* was detected in two *A. mellifera* L. colonies in the Ryazan Region (Russia). Mite identification was supported by COI-based PCR followed by sequencing and BLASTN analysis (NCBI BLASTN, version 2.17.0+; National Center for Biotechnology Information, Bethesda, MD, USA). Because this observation has not yet been formally published, it is reported here as a preliminary unpublished record.

Previous studies have established that *T. mercedesae* reproduction is strongly dependent on the presence of honey bee brood and that the mite has only a short dispersal phase on adult bees [6,11]. Recent studies have also documented the geographic expansion of *T. mercedesae* beyond its historically recognized tropical and subtropical range, including its occurrence in regions with colder seasonal climates [15,26,28]. However, information on the seasonal biology of *T. mercedesae* under these newly occupied climatic conditions remains limited. In particular, it is unclear how infestation changes across seasons in regions with prolonged brood interruption and whether temperature, humidity, and brood availability are associated with these dynamics. Therefore, comparative field data from different climatic regions are needed to understand the persistence and seasonal dynamics of *T. mercedesae* outside its historically recognized range.

The objective of the present study was to investigate the biological characteristics of *T. mercedesae* parasitizing *A. mellifera* L. under non-endemic climatic conditions and to characterize the seasonal dynamics of brood infestation in relation to annual changes in temperature and relative humidity. Improved understanding of these processes will contribute to explaining the ongoing geographic expansion of the parasite and provide a scientific basis for surveillance and prevention strategies aimed at limiting its further spread.

## 2. Material and Methods

### 2.1. Study Sites

The study was conducted between 2022 and 2025 in naturally infested *Apis mellifera* L. apiaries located in three regions representing contrasting climatic conditions in Eurasia: Sochi, Krasnodar Region, Russia (43.453848° N, 39.951575° E; elevation approximately 7 m a.s.l.); Makhachkala, Republic of Dagestan, Russia (42.983333° N, 47.500000° E; elevation approximately 10 m a.s.l.), and Zhetisay, Turkestan Region, Republic of Kazakhstan (40.7763° N, 68.3277° E; elevation approximately 269 m a.s.l.). Site elevations were derived from the Shuttle Radar Topography Mission (SRTM) digital elevation model [29].

Sochi, located on the Black Sea coast, has a humid subtropical climate (Köppen–Geiger Cfa), with a mean annual temperature of 14.7 °C and annual precipitation of approximately 1627 mm for 1991–2020 [30]. Makhachkala, located on the Caspian Sea coast, has a cold semi-arid climate (BSk), with a mean annual temperature of 12.9 °C and annual precipitation of approximately 352 mm [30,31]. Zhetysay has a dry continental climate characterized by pronounced seasonal temperature variation, with a mean annual temperature of approximately 15.0 °C and annual precipitation of approximately 339 mm [30,31]. Climatic characteristics were summarized using the 1991–2020 climatological reference period, corresponding to the current WMO climatological standard-normal period [32].

The surveyed apiaries contained approximately 140 colonies in Sochi, 250 colonies in Makhachkala, and 200 colonies in Zhetisay. Colonies in Sochi maintained brood throughout the year, whereas brood interruption occurred from November to February in Dagestan and from October to February/March in Kazakhstan. At all study sites, colonies were maintained in 16-frame Dadant hives (frame size: 435 × 300 mm). According to information provided by the respective beekeepers, colonies in Sochi were maintained as *A. m. caucasica* Gorb., whereas those in Makhachkala and Zhetisay were maintained as *A. m. carnica* Poll. Bee subspecies identity was not independently verified as part of the present study.

These differences in climatic conditions and seasonal brood availability provided an opportunity to compare the persistence and seasonal dynamics of *T. mercedesae* under contrasting non-endemic environmental conditions.

### 2.2. Climatic Data

Monthly mean air temperature (°C), relative humidity (%), and total monthly precipitation (mm) were obtained from online meteorological databases for each study region throughout the observation period. Meteorological data for Russia were obtained from Meteo9 (https://meteo9.ru/, accessed on 1 March 2026), whereas data for Kazakhstan were obtained from RP5 (https://rp5.ru/, accessed on 1 March 2026). These variables were used to describe seasonal climatic conditions and to compare infestation dynamics among regions.

Because the meteorological stations were located in urbanized areas rather than directly at the study apiaries, the recorded climatic variables may not fully represent apiary-level microclimatic conditions. Therefore, these data were used primarily to characterize regional temporal patterns, and climate–infestation associations were interpreted cautiously in light of this potential exposure misclassification.

### 2.3. Colony Inspection and Assessment of T. mercedesae Infestation

Colonies were inspected monthly throughout the study period. Colony strength and brood abundance were assessed following the standardized methods described in the COLOSS BEEBOOK [33], while examination for *Tropilaelaps* infestation followed the diagnostic recommendations of the World Organization for Animal Health (WOAH) [34]. The presence of *T. mercedesae* was evaluated using two complementary methods. First, all brood frames were visually inspected after the application of smoke and gentle tapping of the frame to facilitate detection of adult mites on the comb surface. This procedure was used for routine surveillance of colony infestation. Second, brood infestation was quantified by examining approximately 100 sealed worker brood cells. A comb section (approximately 3 × 15 cm) containing purple-eyed pupae was removed and cooled at 4–6 °C for 2–3 h before uncapping. Cooling reduced mite movement and enabled reliable counting of adult mites and immature stages within brood cells. Monthly brood infestation is expressed as the percentage of examined sealed brood cells infested with *T. mercedesae*.

Routine acaricidal treatments against *V. destructor* were conducted annually at all three study sites (Sochi, Makhachkala, and Zhetisay). The management programs included fluvalinate-based treatments (Fumisan®, API-SAN LLC, Moscow Region, Russia) in spring, 85% formic acid (Muravinka®, Apisfera 2000 LLC, Moscow, Russia) applied after honey harvest (July–August; three applications at 7-day intervals), and amitraz-based treatments (Bipin-T®, Agrobioprom JSC, Moscow, Russia) in autumn (September–October), when brood abundance was reduced. These treatments were part of routine apiary management and were not experimental treatments in the present study. *V. destructor* infestation levels were not quantitatively assessed; therefore, site- or colony-specific *Varroa* infestation-load data were not available for analysis.

For the preliminary Ryazan record, mite identity was additionally assessed by PCR amplification of the mitochondrial cytochrome *c* oxidase subunit I (COI) region according to the EURL/ANSES protocol [35], followed by sequencing of the PCR products and sequence comparison using BLAST version 2.17.0+ [36].

### 2.4. Morphometric Analysis

Adult female *T. mercedesae* mites were preserved in 95% ethanol (JSC RFK, Oryol Region, Russia) immediately after collection. Two hundred females from each study region were randomly selected for morphometric analysis. Measurements were performed using an MBS-10 stereomicroscope equipped with a digital camera (LZOS, Lytkarino, Moscow Region, Russia). Six diagnostic morphometric characters were measured following WOAH guidelines [34] and previously published protocols: dorsal shield length; dorsal shield width; epigynial shield length; epigynial shield width; ventrianal shield length; ventrianal shield width [37]. Morphometric measurements were used to compare regional populations and evaluate geographic variation among non-endemic populations.

### 2.5. Detection of Honey Bee Viruses by Conventional RT-PCR

For viral analysis, 30 adult female *T. mercedesae* collected from a single colony were combined into one pooled sample, which was considered the experimental unit. Eight pooled samples, each representing a different colony, were analyzed: two from Sochi, four from Makhachkala, and two from Zhetisay. Samples were collected from colonies showing clinical signs consistent with DWV infection. No a priori sample-size calculation was performed because this analysis was conducted as preliminary exploratory screening.

The presence of honey bee viruses in *T. mercedesae* was assessed by reverse-transcription polymerase chain reaction (RT-PCR). Total RNA was extracted from ethanol-preserved mites using a modified guanidinium isothiocyanate-based extraction procedure [38,39]. Mite samples were homogenized with quartz sand in 500 µL of guanidinium thiocyanate buffer containing 3 M guanidinium thiocyanate, 0.025 M sodium citrate, and 0.5% Triton X-100 (Sigma-Aldrich, St. Louis, MO, USA), and incubated at 65 °C for 40 min in a Gnom thermoblock (DNA-Technology, Moscow, Russia). After cooling, 500 µL of chloroform (Sigma-Aldrich, St. Louis, MO, USA) was added, and the samples were vigorously mixed and centrifuged at 13,000 rpm for 5 min using a Microspin 12 mini-centrifuge (Biosan, Riga, Latvia). The aqueous phase was transferred to a new tube, mixed with an equal volume of chloroform, and centrifuged again at 13,000 rpm for 5 min. The resulting aqueous phase was transferred to a clean microcentrifuge tube, mixed with 0.1 volume of 3 M sodium acetate (pH 5.2; Sigma-Aldrich, St. Louis, MO, USA) and two volumes of 96% ethanol, and incubated overnight at −20 °C to precipitate RNA.

Following overnight precipitation, 800 µL of the suspension was transferred to a clean microcentrifuge tube and centrifuged at 13,000 rpm for 15 min. The supernatant was removed, and the pellet was washed with 200 µL of 70% ethanol and centrifuged for 30 s. After removal of the supernatant, the washing procedure was repeated using 96% ethanol. The pellet was dried at 65 °C for 40 min in a Gnom thermoblock (DNA-Technology, Moscow, Russia) and resuspended in 4 µL of deionized water. RNA concentration and purity were assessed using a UNICO 2802(S) spectrophotometer (United Products & Instruments, Inc., Dayton, NJ, USA).

First-strand cDNA was synthesized from 50 ng of total RNA using M-MLV reverse transcriptase (SibEnzyme, Novosibirsk, Russia) and random decanucleotide primers (Eurogen, Moscow, Russia). Reverse transcription was performed using individual reagents rather than a commercial cDNA synthesis kit. The RNA was mixed with 1 μL of 20 μM random decanucleotide primers, denatured at 95 °C for 5 min in a Gnom thermoblock (DNA-Technology, Moscow, Russia), and immediately chilled on ice. The reverse-transcription reaction was prepared to a final volume of 10 μL by adding 1 μL of 10× reverse-transcription buffer, 1 μL of 4 mM dNTP mixture, 1 μL of M-MuLV reverse transcriptase (200 U/μL) (SibEnzyme, Novosibirsk, Russia), and deionized water. The reaction mixture was incubated at 37 °C for 70 min in the Gnom thermoblock for first-strand cDNA synthesis. Following reverse transcription, the reaction mixture was diluted to 30 µL with deionized water, supplemented with 0.1 volume of sodium acetate and two volumes of 96% ethanol, and incubated overnight at −20 °C. The samples were then centrifuged at 13,000 rpm for 15 min, and the supernatant was removed. The pellet was washed with 300 µL of 70% ethanol and centrifuged for 30 s. After removal of the supernatant, the washing procedure was repeated using 96% ethanol. The pellet was dried at 65 °C for 40 min and finally resuspended in 12 µL of deionized water.

Seven honey bee viruses were screened: Deformed wing virus (DWV), Acute bee paralysis virus (ABPV), Chronic bee paralysis virus (CBPV), Kashmir bee virus (KBV), Israeli acute paralysis virus (IAPV), Black queen cell virus (BQCV), and Sacbrood virus (SBV).

Reference nucleotide sequences of the target viruses were retrieved from the National Center for Biotechnology Information (NCBI) database. The corresponding accession numbers are provided in Supplementary Table S2. Multiple sequence alignments were performed using AliBee Multiple Alignment Release 3.0 (http://www.genebee.msu.su/, accessed on 1 February 2026) to identify conserved genomic regions suitable for primer design. Primers (20–25 bp) were designed from conserved regions, preferentially beginning and ending with guanine (G) or cytosine (C) residues to improve amplification efficiency. Candidate primer pairs were evaluated for annealing temperature compatibility and sequence specificity. Primer specificity was verified using NCBI BLASTN (https://www.ncbi.nlm.nih.gov/, accessed on 1 February 2026) to minimize non-specific amplification. Primer pairs exhibiting high predicted specificity and similar annealing temperatures were synthesized by Eurogen (Moscow, Russia). The primer sequences used in this study are presented in Table 1; more information can be found in Supplementary Table S2.

Conventional PCR amplification was performed in a 10 μL reaction mixture containing 2 μL of cDNA, 1× PCR buffer, 0.2 mM of each dNTP, 1 μM each of the forward and reverse primers, 0.75 U of Taq DNA polymerase (SibEnzyme, Novosibirsk, Russia), and nuclease-free water. Amplification was carried out using a Tertsik thermal cycler (DNA Technology, Moscow, Russia) under the following cycling conditions: initial denaturation at 95 °C for 5 min; 35 cycles of 95 °C for 30 s, 42 °C for 30 s, and 72 °C for 30 s; followed by a final extension at 72 °C for 8 min.

PCR products were separated by electrophoresis in 6% native polyacrylamide gels, stained with ethidium bromide, and visualized under ultraviolet illumination using a 100-bp DNA ladder (SibEnzyme, Novosibirsk, Russia) as the molecular size marker. Samples producing amplicons of the expected size were considered positive. Representative PCR products were excised from the gel, purified, and subjected to Sanger sequencing (Beagle LLC, St. Petersburg, Russia) to confirm the identity of the amplified viral fragments.

### 2.6. Statistical Analysis

All statistical analyses were performed using XLSTAT Advanced, version 2025.1.3 (Addinsoft, Paris, France). Differences in morphometric characters among regional *T. mercedesae* populations were evaluated using one-way analysis of variance (ANOVA). Least-square means (LS means) were compared using Tukey’s honestly significant difference (HSD) post hoc test. Statistical significance was accepted at *p* < 0.05. To evaluate overall morphological differentiation among populations, multivariate analysis of variance (MANOVA) was performed using the six morphometric variables. Canonical discriminant analysis (CDA) was subsequently applied to visualize population separation and identify the morphological characters contributing most strongly to regional discrimination. Classification accuracy was evaluated by leave-one-out cross-validation. Monthly infestation dynamics were compared descriptively among regions using temporal trends in brood infestation together with corresponding monthly climatic variables (air temperature, relative humidity, and precipitation). Virus detection results are summarized as presence/absence for each virus in each regional population because of the limited sample size available for virological analyses. Pearson correlation coefficients were calculated separately for each study region to assess relationships among colony strength (frames covered with bees), brood abundance (frames), *Tropilaelaps* brood infestation (%), average air temperature (°C), relative humidity (%), monthly precipitation (mm), and month. All graphs were generated using XLSTAT.

## 3. Results

*Tropilaelaps mercedesae* was detected annually at all three study locations during the observation period. Following the initial records, the mite was repeatedly detected in colonies in Sochi and Makhachkala (Russia) and Zhetisay (Kazakhstan), demonstrating recurrent occurrence across successive years. Thus, *T. mercedesae* was not restricted to a single detection event at any of the investigated locations.

### 3.1. Climatic Differentiation Among Study Regions in 2025

**Figure 2:**
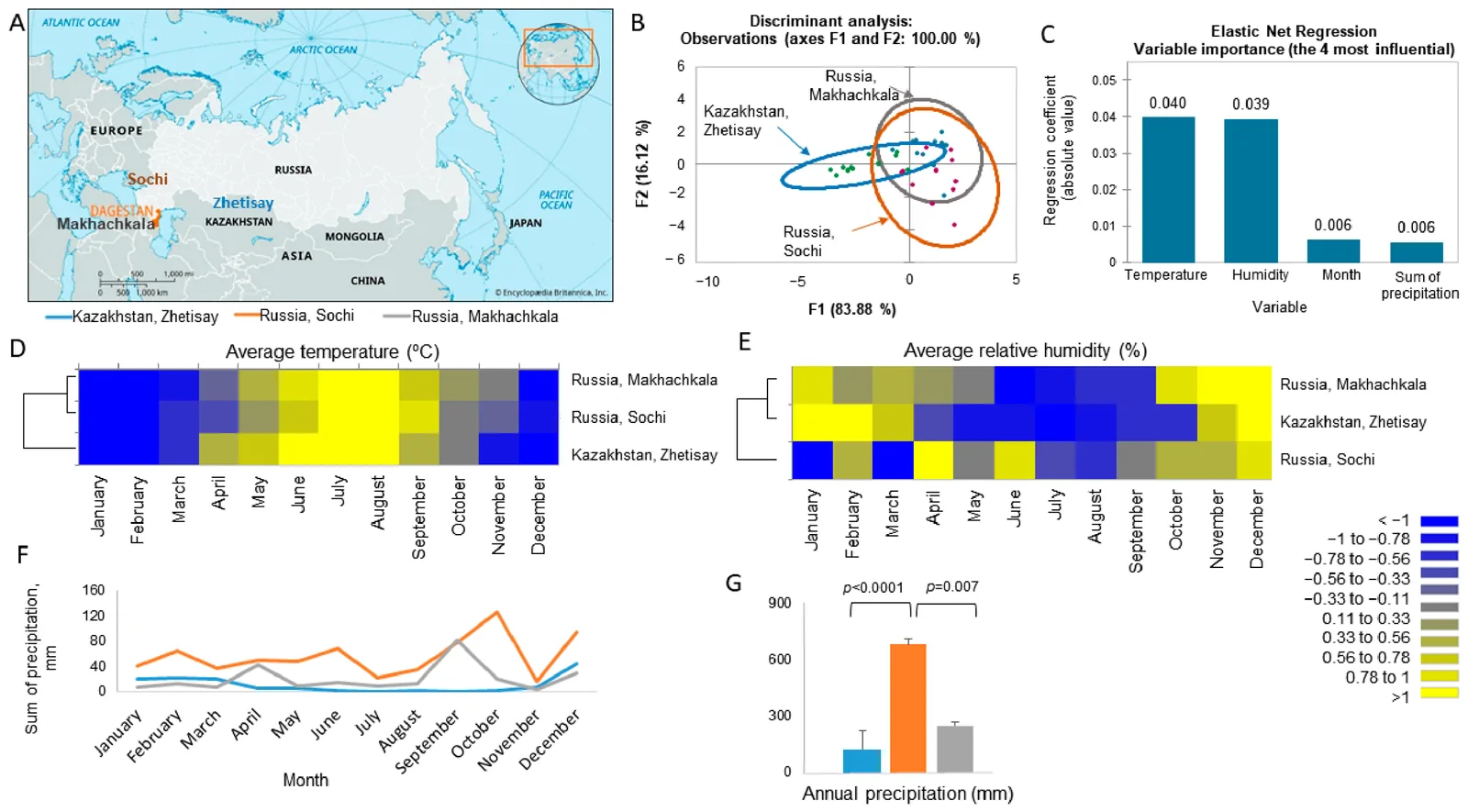
Distinct climatic regimes characterize the study regions across a Eurasian environmental gradient in 2025. (**A**) Geographic locations of the study regions in Sochi (Russia), Makhachkala (Russia), and Zhetisay (Kazakhstan). (**B**) Canonical discriminant analysis based on average temperature, relative humidity, and precipitation, demonstrating multivariate climatic differentiation among regions. Points represent individual observations, colors distinguish the three study regions, and colored ellipses delineate the regional groupings in the discriminant-analysis plot. (**C**) Elastic Net Regression variable importance showing the relative contribution of climatic predictors to regional climatic separation. (**D**) Standardized heatmap of monthly temperature patterns. (**E**) Standardized heatmap of monthly relative humidity patterns. (**F**) Monthly precipitation dynamics across the three regions during 2025. (**G**) Regional comparison of cumulative annual precipitation, with significant pairwise differences indicated above bars. Heatmap colors represent centered and standardized values (z-scores), highlighting relative seasonal deviations within each climatic variable and facilitating comparison of climatic patterns among regions.

The three study regions represented significantly different climatic environments, as demonstrated by MANOVA, canonical discriminant analysis, and Elastic Net Regression (Figure 2). Temperature and relative humidity accounted for most of the climatic differentiation among regions, whereas precipitation contributed comparatively little. Zhetisay (Kazakhstan) was characterized by the lowest humidity and precipitation and the greatest annual temperature amplitude, whereas Sochi (Russia) exhibited the highest annual precipitation and Makhachkala (Russia) maintained consistently high humidity. These results demonstrate that the studied honey bee and mite populations developed under markedly different environmental conditions.

### 3.2. Interannual Climatic Stability

Comparison of climatic variables between 2024 and 2025 showed that the general climatic characteristics of each region remained stable. Zetisay (Kazakhstan) exhibited significant interannual differences in temperature, whereas Sochi (Russia) showed differences in humidity (Supplementary Figure S1). In contrast, no significant interannual variation was detected in Mahachkala (Russia). Overall, these results indicate that regional climatic differences were substantially greater than year-to-year climatic variation.

### 3.3. Seasonal Dynamics of Colony Development and T. mercedesae Infestation

**Figure 3:**
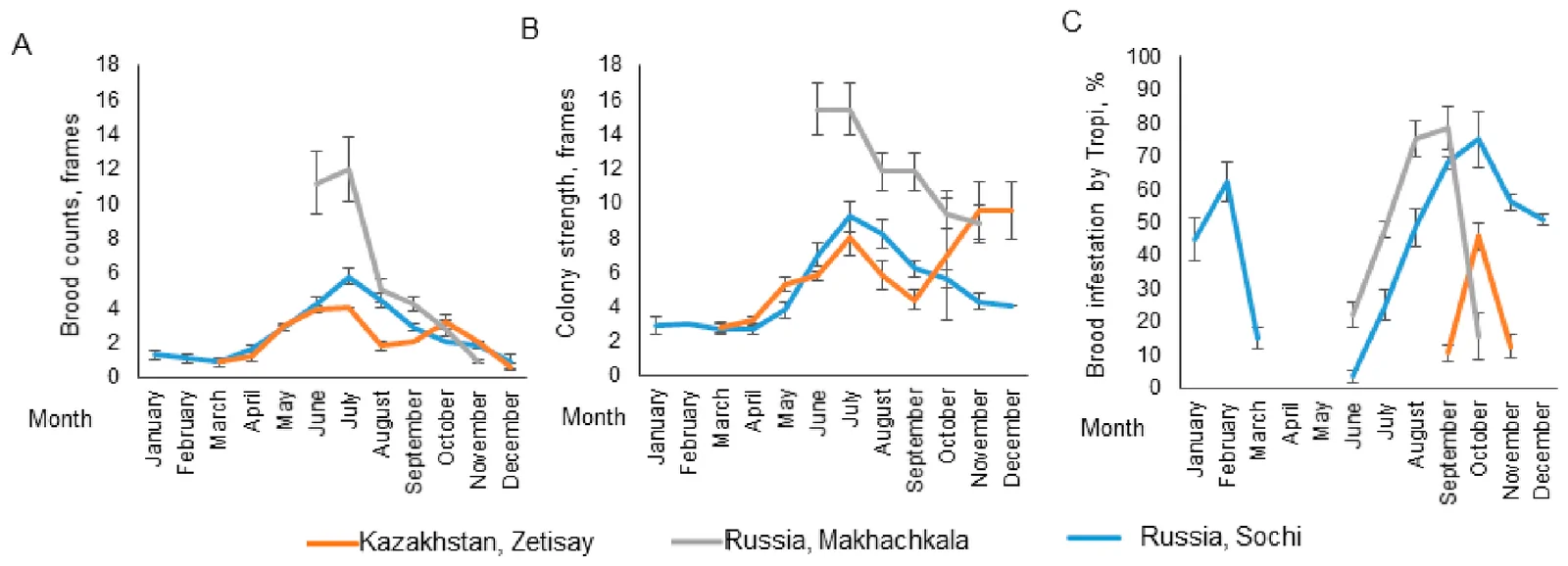
Seasonal dynamics of honey bee colonies and *T. mercedesae* infestation in three regions during 2025. (**A**) Brood abundance (frames). (**B**) Colony strength (frames covered with bees). (**C**) Brood infestation by *T. mercedesae* (%). Values are presented as mean ± SE. Orange lines indicate Zhetisay (Kazakhstan), grey lines indicate Makhachkala (Russia), and blue lines indicate Sochi (Russia).

Despite the stable establishment of *T. mercedesae* in all study regions, seasonal infestation patterns differed markedly (Figure 3). In Sochi (Russia), honey bee colonies maintained brood throughout the year. Nevertheless, infestation declined during late winter and was not detected in April and May despite the continued presence of brood. *T. mercedesae* reappeared in June, and infestation increased progressively towards autumn. In Mahachkala (Russia), the mite was first detected in June. Population growth accelerated throughout summer, reaching maximum infestation during August–September, followed by a gradual decline until mites were no longer detected in November. In Zhetisay (Kazakhstan), detectable infestation was restricted to a short period during autumn, first appearing in September, peaking in October, and declining again in November.

Thus, although *T. mercedesae* persisted in all three regions, the duration of detectable infestation differed substantially, suggesting strong regional differences in seasonal population development.

### 3.4. Relationships Between Climate, Colony Development, and Infestation

**Figure 4:**
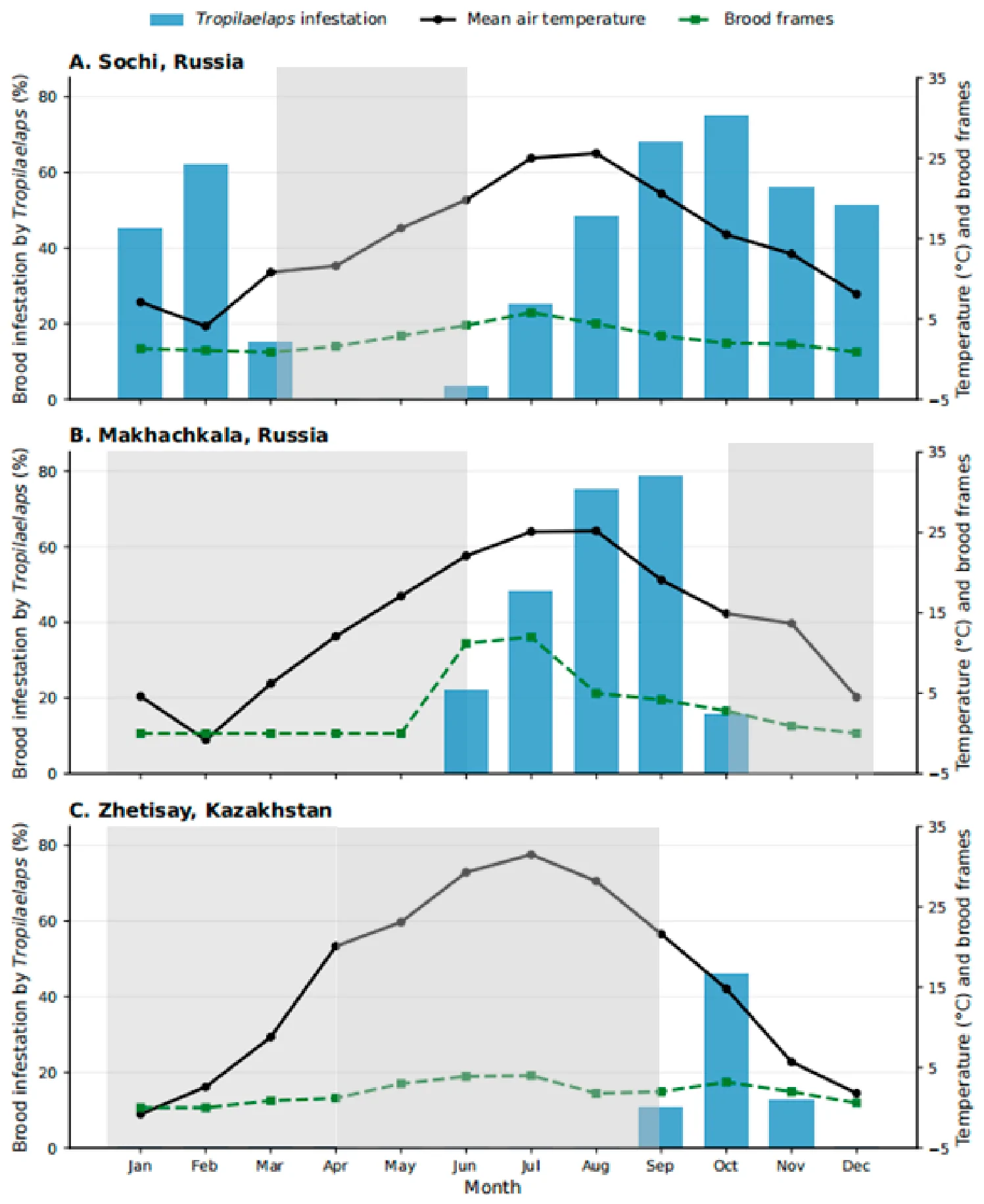
Regional seasonal dynamics of *Tropilaelaps mercedesae* infestation in relation to brood development and ambient temperature in honey bee (*Apis mellifera* L.) colonies (2025). Monthly mean brood infestation by *T. mercedesae* is shown as blue bars (left *y*-axis). Mean monthly air temperature (black solid line) and brood development expressed as the number of brood frames (green dashed line) are plotted on the right *y*-axis. Panels represent (**A**) Sochi (Russia), (**B**) Makhachkala (Russia), and (**C**) Zhetisay (Kazakhstan). Light grey shading indicates the period when no infestation was detected in the brood.

Regional comparisons revealed that seasonal infestation dynamics were closely associated with brood availability rather than with individual climatic variables alone (Figure 4). In all three regions, *T. mercedesae* populations increased only after brood production became established and declined when brood production decreased or ceased. However, the timing and duration of detectable infestation differed substantially among regions.

In Sochi (Russia), colonies maintained brood throughout the year, yet mites remained undetectable from February to May before infestation increased progressively during summer and reached its maximum in autumn. In Makhachkala (Russia), brood production resumed in early summer and was accompanied by a rapid increase in mite infestation, which peaked during August–September. In contrast, infestation in Zhetisay (Kazakhstan) was restricted to a short period during late summer and early autumn despite favorable summer temperatures.

Pearson correlation analysis supported these regional differences (Supplementary Figure S2). The strongest relationships were observed in Makhachkala (Russia), where infestation was positively associated with brood abundance (r = 0.583) and air temperature (r = 0.718), whereas humidity showed a negative association (r = −0.585). Correlations were considerably weaker in Sochi (Russia) and Zhetisay (Kazakhstan), suggesting that climatic variables alone cannot fully explain regional variation in infestation dynamics.

Taken together, these observations indicate that regional climatic conditions primarily influence *T. mercedesae* populations indirectly through their effects on colony brood phenology rather than acting as independent drivers of infestation.

### 3.5. Morphometric Differentiation of Regional Populations

**Figure 5:**
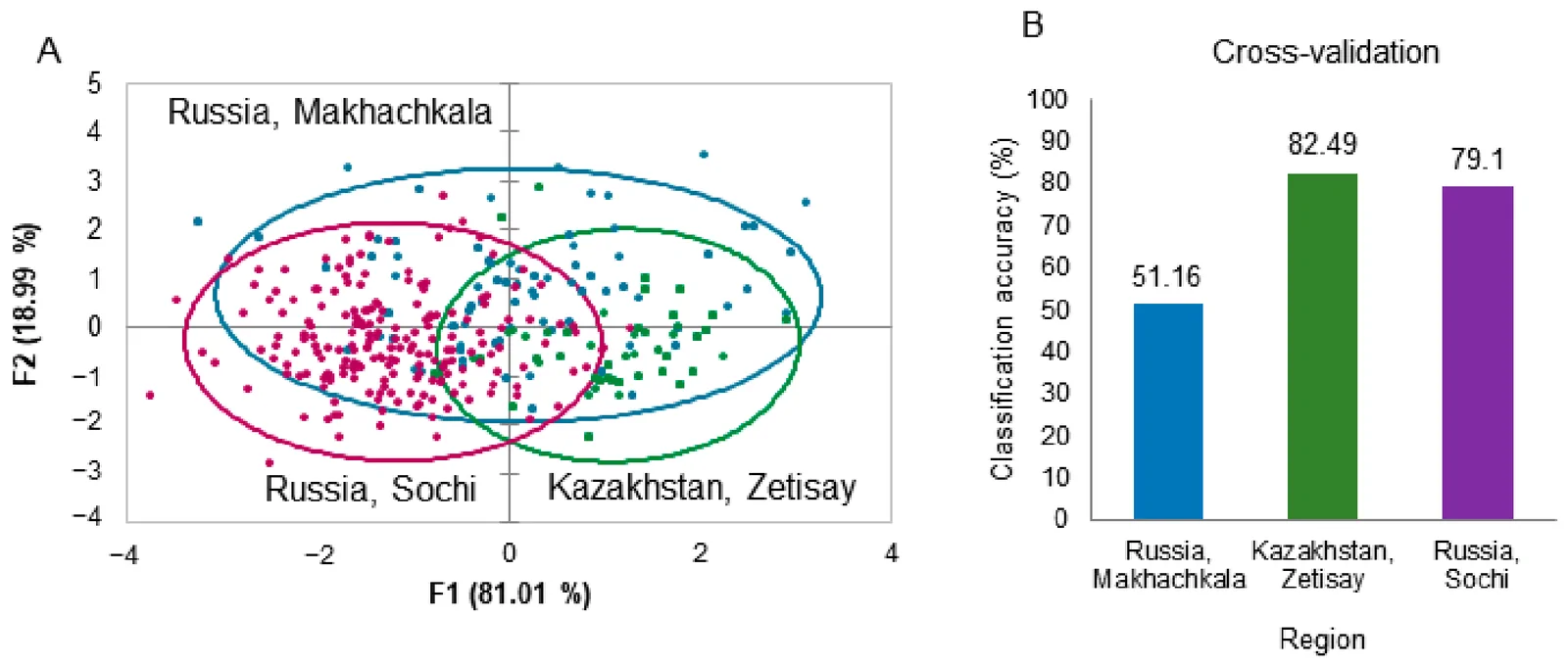
Morphometric differentiation of *T. mercedesae* populations from Zhetisay (Kazakhstan, *n* = 23), Makhachkala (Russia; *n* = 29), and Sochi (Russia; *n* = 23). (**A**) Discriminant analysis based on six morphometric traits. The first two canonical functions explained 100% of the total variation (F1 = 81.01%, F2 = 18.99%). (**B**) Cross-validation classification accuracy (%) for each population.

Because infestation dynamics differed markedly among regions, morphometric variation among *T. mercedesae* populations was investigated (Figure 5). Canonical discriminant analysis demonstrated clear separation among the Zhetisay (Kazakhstan), Makhachkala (Russia), and Sochi (Russia) populations. The first canonical function explained 81.0% of the total variation, and the overall classification accuracy was 70.9%, indicating measurable regional differentiation. Among the six examined morphometric characters (Supplementary Figure S3), body width (R^2^ = 0.318), body length (R^2^ = 0.243), and abdominal shield length (R^2^ = 0.236) exhibited the strongest regional differentiation based on one-way ANOVA (Supplementary Table S3).

### 3.6. Virus Carriage by Tropilaelaps mercedesae

Molecular screening of *T. mersedesae* detected RNA of honey bee-associated viruses in the examined mite samples. Seven target viruses were screened by reverse-transcription polymerase chain reaction (RT-PCR). Viral RNA detection was based on PCR products of the expected amplicon size following amplification with virus-specific primers. For DWV and CBPV, amplicon identity was additionally confirmed by nucleotide sequencing.

**Figure 6:**
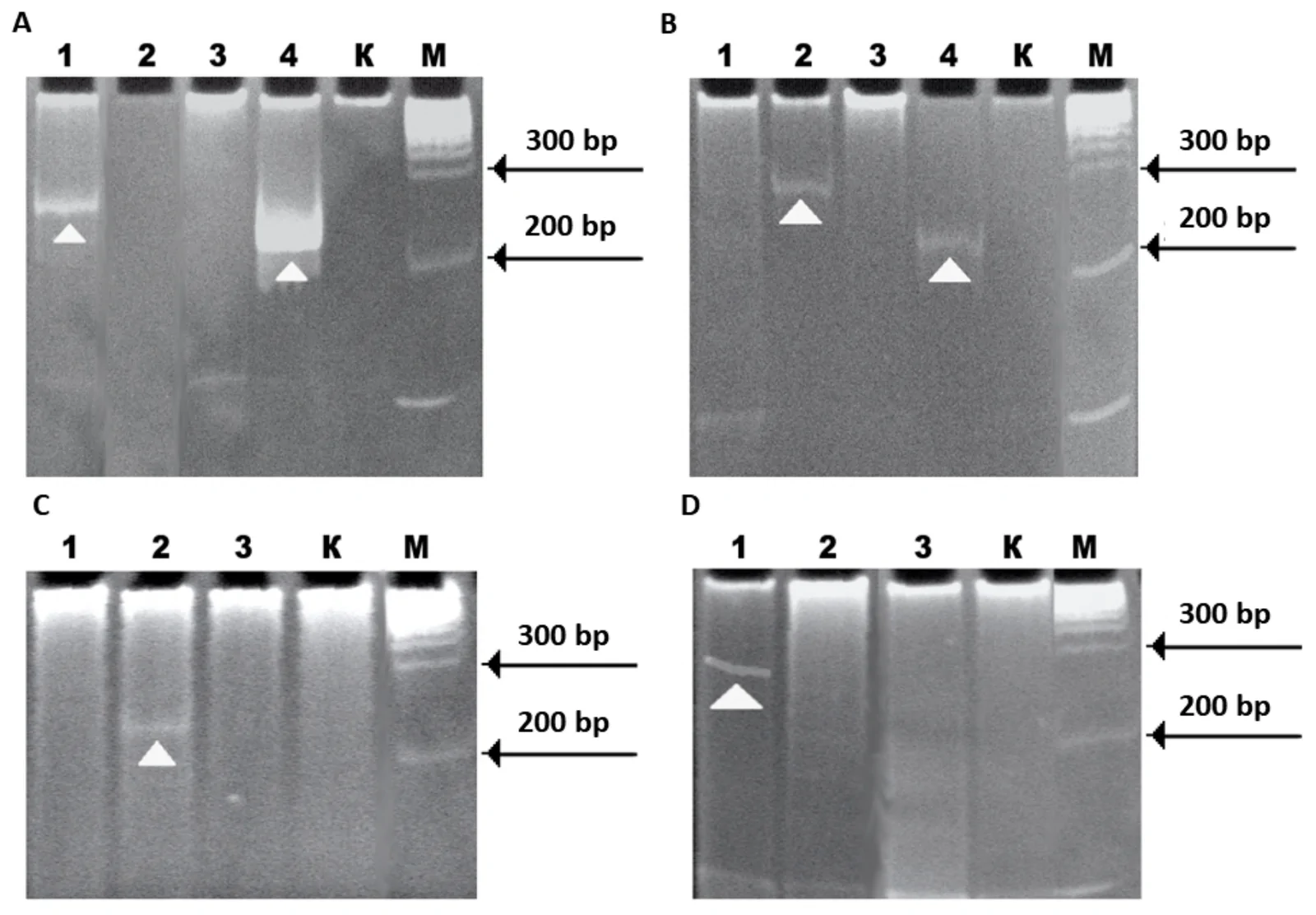
Representative RT-PCR electropherograms showing amplification of target honey bee virus sequences from *T. mersedesae* samples collected from different geographic populations. Panels (**A**,**B**) show amplification products obtained with primers specific for SBV, ABPV, BQCV, and DWV (lanes 1–4, respectively). Panels (**C**,**D**) show amplification products obtained with primers specific for KBV, CBPV, and IAPV (lanes 1–3, respectively). Lane K represents the negative PCR control, and lane M denotes the 100-bp DNA ladder (SibEnzyme). White arrowheads indicate the specific amplification products corresponding to the expected amplicon sizes.

Four viruses were detected in the analyzed samples: Deformed wing virus (DWV), Acute bee paralysis virus (ABPV), Chronic bee paralysis virus (CBPV), and Kashmir bee virus (KBV) (Table 2, Figure 6). DWV was the only virus detected in mite populations from all three geographic regions and was the most frequently detected virus overall. ABPV and CBPV were detected in samples from Sochi and Dagestan but were not detected in samples from Kazakhstan. KBV was detected only in the Dagestan population. Israeli acute paralysis virus (IAPV), Black queen cell virus (BQCV), and Sacbrood virus (SBV) were not detected in any of the examined samples.

The pattern of viral RNA detection differed among geographic regions. Samples from Makhachkala were positive for the greatest number of target viruses, whereas samples from Zetizay were positive only for DWV. Samples in which none of the seven target viruses were detected were found in each of the three regions (Table 2). Overall, DWV was the only target virus consistently detected across all three geographic regions.

## 4. Discussion

### 4.1. Factors Potentially Contributing to the Persistence of Tropilaelaps mercedesae in Temperate Eurasia

The present study documents the repeated occurrence of *T. mercedesae* in *A. mellifera* colonies under non-endemic climatic conditions in southern Russia and southeastern Kazakhstan over multiple years. Unlike isolated reports, the consistent detection of the mite in geographically separated populations suggests that *T. mercedesae* is capable of persisting under temperate environmental conditions and is not restricted to sporadic introductions.

Historically, *T. mersedesae* were considered parasites confined to tropical and subtropical regions of Asia, where continuous brood production allows uninterrupted reproduction [4,22,24]. Consequently, the absence of brood during winter was regarded as one of the principal barriers preventing their establishment in temperate climates. Early literature also identified *T. clareae* as the predominant species responsible for economically important infestations of honey bee colonies [4,20,23]. However, the taxonomic revision of Anderson and Morgan [2] demonstrated that many populations previously identified as *T. clareae* actually represented distinct species, particularly *T. mercedesae*. This revision substantially changed current understanding of the distribution, host associations, and epidemiological significance of *Tropilaelaps* species.

The recent westward expansion of *Tropilaelaps* has also been recognized by the European Union Reference Laboratory for Bee Health, which reported its occurrence in territories close to the European Union and highlighted climatic conditions potentially favorable for its survival [14]. Our observations agree with recent reports documenting *T. mercedesae* in Uzbekistan, Georgia, and southern Russia [17,18,40]. Together, these studies indicate that the known distribution of *T. mercedesae* now extends considerably farther west than previously recognized. Although improvements in molecular diagnostics have undoubtedly increased the accuracy of species identification, they alone are unlikely to explain the growing number of detections reported during the last decade. The increasing occurrence of *T. mercedesae* outside its historically recognized range is more likely the result of several interacting factors, including international movement of managed honey bee colonies and commercial bee packages, together with environmental changes that may extend brood-rearing periods and increase opportunities for mite reproduction.

One of the most notable findings of the present study is the repeated detection of *T. mercedesae* in regions where honey bee colonies normally undergo seasonal winter brood interruption. Because *Tropilaelaps* spp. reproduce exclusively within sealed brood cells and adult mites survive only for limited periods in the absence of brood [2,22,25], brood interruption has traditionally been considered a natural barrier limiting establishment in temperate climates. Nevertheless, the repeated annual occurrence observed in this study indicates that brood interruption alone does not necessarily prevent the long-term persistence of *T. mercedesae* populations under temperate conditions.

The biological mechanisms responsible for this persistence remain uncertain. Several non-exclusive explanations may account for the observed pattern. First, repeated annual introductions through the movement of infested bee colonies or commercial bee packages could contribute to the continued presence of the mite. Second, *T. mercedesae* may possess greater ecological plasticity than previously recognized, enabling survival under environmental conditions outside its historically described climatic range. Third, progressively warmer winters and longer brood-rearing periods associated with climate change may increase opportunities for population persistence and reproduction. Because the present study was observational, it cannot distinguish among these hypotheses or determine their relative contributions.

Future studies integrating long-term field monitoring, molecular epidemiology, controlled overwintering experiments, and climatic analyses are needed to clarify the mechanisms responsible for the persistence and further spread of *T. mercedesae* in temperate regions. Such information will be essential for improving risk assessment, strengthening surveillance programs, and developing effective management strategies for this emerging parasite of *A. mellifera* L.

### 4.2. Regional Climatic Differences Influence Seasonal Infestation Rather than Establishment

One of the principal findings of this study is that *T. mersedesae* successfully established populations under three contrasting climatic regimes, whereas the seasonal dynamics of infestation differed substantially among regions. Climatic analyses (Figure 2) confirmed pronounced differences in temperature and relative humidity among Sochi (Russia), Makhachkala (Russia), and Zhetisay (Kazakhstan). Nevertheless, the mite became established in all regions, suggesting that regional climate did not determine whether establishment occurred, but rather influenced the seasonal timing and duration of detectable infestation.

Seasonal infestation dynamics closely followed regional differences in colony development (Figure 4). Because *Tropilaelaps* spp. reproduces exclusively within sealed brood cells [2,22], regional variation in brood availability provide a biologically plausible explanation for the observed differences in infestation dynamics [21]. In Makhachkala (Russia) and Zhetisay (Kazakhstan), detectable mite populations emerged only after brood production resumed and declined towards the end of the brood-rearing season. These observations are consistent with the brood-dependent reproductive biology of *T. mersedesae*, although additional factors are likely to contribute to seasonal population dynamics.

An interesting exception was observed in Sochi, where brood was present throughout the year but *T. mersedesae* remained undetectable from February to May before populations increased during summer and autumn. This pattern indicates that brood availability alone cannot fully explain seasonal population dynamics. A similarly complex relationship between brood availability and *Tropilaelaps* abundance has been reported under Indian conditions, where seasonal mite incidence did not increase proportionally with brood abundance and was negatively correlated with brood area [41]. Furthermore, failure to detect *Tropilaelaps* should not necessarily be interpreted as complete absence of the mite, because detection probability can vary substantially among surveillance methods, particularly at low infestation levels [42].

Other factors, including colony demography, post-winter mite population size, host–parasite interactions, or additional environmental variables not measured in the present study, may also influence seasonal detectability [2,22]. These observations suggest that the ecology of *T. mercedesae* in recently colonized temperate regions is more complex than previously assumed [2,12].

Relative humidity showed a moderate negative correlation with brood infestation in Makhachkala (r = −0.585), whereas the relationship was weak in Zhetisay (r = −0.169) and negligible in Sochi (r = 0.020), according to the criteria of Schober et al. [43] (Supplementary Figure S2). The lack of a consistent relationship among regions suggests that relative humidity alone is unlikely to explain infestation dynamics. In Makhachkala, humidity also covaried with temperature and brood abundance, making it difficult to separate its independent effect from other seasonal factors. Furthermore, ambient meteorological conditions do not directly represent the microclimate experienced by mites within the colony, because honey bees actively regulate brood-nest conditions, although external climatic extremes can influence within-hive conditions [44]. Because the correlation analysis was based on observations from a single year (2025), it cannot establish causal relationships between climatic variables and mite infestation. Multi-year monitoring is required to determine whether these associations are reproducible across years and to better distinguish climatic effects from seasonal changes in colony and brood dynamics.

Because the present analyses are based on field observations and exploratory correlations, they should be interpreted as evidence of ecological associations rather than direct causal relationships between climatic variables and parasite population dynamics.

### 4.3. Morphological Differentiation Following Establishment

Morphometric analysis demonstrated measurable differentiation among geographically separated *T. mersedesae* populations (Figure 5). Canonical discriminant analysis separated mites from the three study regions with an overall classification accuracy of 70.9%, indicating statistically detectable morphological variation among regional populations. Body width showed the strongest regional effect (R^2^ = 0.318), whereas anal shield measurements exhibited comparatively small regional effects (R^2^ = 0.037–0.051), indicating that geographic differentiation was concentrated in overall body morphology rather than all measured structures. Morphological variability has previously been reported among geographically separated populations of *Tropilaelaps* spp. and other parasitic mites and may reflect differences in environmental conditions, host populations, or population history [2]. However, the present study cannot determine whether the observed differences resulted from founder effects following introduction, phenotypic plasticity under contrasting environmental conditions, or local genetic adaptation. Consequently, the observed morphometric variation should be interpreted as evidence of regional population differentiation rather than direct evidence of evolutionary adaptation. Integrating morphometric analyses with population genomic approaches and broader geographic sampling will be necessary to determine the relative contributions of demographic history, phenotypic plasticity, and local adaptation during the ongoing expansion of *T. mercedesae*.

### 4.4. Virus Carriage by Tropilaelaps mercedesae

Another important finding was the detection of honey bee virus RNA in *T. mersedesae* samples from all investigated regions. Four of the seven screened viruses (DWV, ABPV, CBPV, and KBV) were detected, whereas BQCV, SBV, and IAPV were not identified. DWV was the only virus consistently detected in mite samples from all three regions, supporting previous studies reporting an association between DWV and *Tropilaelaps* spp. [45,46,47].

The pattern of viral RNA detection differed among geographic populations. Mite samples collected in Makhachkala were positive for the greatest number of screened viruses, whereas samples from Zhetisay were positive only for DWV. Although based on a limited number of samples, these observations demonstrate geographic variation in the occurrence of the screened viral RNAs in *T. mersedesae* under the investigated non-endemic conditions.

However, detection of viral RNA in *T. mercedesae* does not demonstrate viral replication in the mite or transmission of these viruses to honey bees. Viral loads and paired bee–mite samples were not analyzed, and *V. destructor* infestation was not quantified in the investigated colonies; therefore, its potential contribution as an alternative source of viral infection cannot be excluded. Further studies combining quantitative viral-load assessment, analysis of viral replication, paired bee–mite sampling, and simultaneous monitoring of *V. destructor* are required to determine the potential epidemiological and vector role of *T. mercedesae*.

## 5. Conclusions

Our study shows that *T. mercedesae* was repeatedly detected across contrasting climatic regions of southern Russia and Kazakhstan. Infestation was strongly seasonal and generally followed brood availability, although the timing and strength of this relationship differed among regions. Importantly, the mite was detected repeatedly across years even in regions with seasonal brood interruption, indicating that such climatic conditions do not prevent its persistence.

Morphometric analysis showed differences among the three geographic mite populations, suggesting regional phenotypic variation. Preliminary viral screening detected RNA of several honey bee-associated viruses in pooled *T. mercedesae* samples; however, these results do not demonstrate viral transmission by the mite.

The temporal association between honey bee imports from Uzbekistan, including transport through Kazakhstan, and the first regional detections suggests that managed bee movement may represent a possible pathway of introduction. However, the source population and invasion route remain unconfirmed. Overall, our findings demonstrate the need for continued surveillance of *T. mercedesae*, including during periods of low brood availability, and for monitoring its further geographic expansion.

## Figures and Tables

**Table 1:** Virus-specific primers.

Target	5′-3′	Amplicon, bp
ABPV	F: TGTGATGTGGGTAGATACATT R: CATAATGCAAACATTCAAAGATC	300
BQCV	F: GACGAAAGGAAGCCTAAACATAA R: GGAAGAGACTTGCACCATTG	420
CBPV	F: GGATGGGAAACGGAAATCATCCCGG R: GACTGCGAGAGGGGTATGTTGTACTG	209
DWV	F: GATATGTGGAGTGTTGAAACATCTAC R: CATTCAATCAAAACATTTCTACGCCG	224
SBV	F: GGTAGAAGCTGG(A/G)GATGATTTTGAG R: CTCGCTC(T/A)GGAGTTACTACTTTTCC	257
KBV	F: CGTGGATATGCTCAGGAGCGG R: AGGTATAGTATTTCGGAACGCCGC	262
IAPV	F: CAGTGATGAACTGTATAACTCATC R: GTAGAGGTTATTATCCGAAATACATG	276

**Table 2:** Detection of honey bee virus RNA in *Tropilaelaps mercedesae* samples collected from three geographic populations.

Virus	Sochi (Russia) (*n* = 2)	Makhachkala (Russia) (*n* = 4)	Zetisay (Kazakhstan) (*n* = 2)
Samples negative for all target viruses	1	1	1
DWV	Detected	Detected	Detected
ABPV	Detected	Detected	Not detected
CBPV	Detected	Detected	Not detected
KBV	Not detected	Detected	Not detected
BQCV	Not detected	Not detected	Not detected
SBV	Not detected	Not detected	Not detected
IAPV	Not detected	Not detected	Not detected

*n* indicates the number of pooled mite samples analyzed; each pooled sample comprised 30 adult female mites collected from one colony. “Detected” indicates detection in at least one pooled sample from the respective region.

## Data Availability

The original contributions presented in this study are included in the article. Further inquiries can be directed to the corresponding authors.

## Supplementary Materials

The following supporting information can be downloaded at https://www.mdpi.com/article/10.3390/insects17090964/s1, Figure S1: Interannual comparison of monthly climatic conditions in Kazakhstan (Zetisay), Russia (Makhachkala and Sochi), between 2024 and 2025; Figure S2: Pearson correlation matrices for honey bee colony parameters, *Tropilaelaps* infestation, and climatic variables in Kazakhstan (A), Russia—Makhachkala (B), and Russia—Sochi (C) during 2025; Figure S3: Morphometric measurements of *Tropilaelaps mercedesae*; Table S1: Published records used to construct the geographic distribution map of *Tropilaelaps* spp. infestations in *Apis mellifera* colonies; Table S2: Honey bee virus sequences retrieved from the NCBI GenBank database and used for primer design and sequence comparison; Table S3: Morphometric characteristics of adult female *Tropilaelaps mercedesae* from three geographic populations.
