# Supplementary material for: Persistence, Seasonal Dynamics, and Regional Differentiation of the Parasitic Mite Tropilaelaps mercedesae in Apis mellifera L. Colonies Under Non-Endemic Climatic Conditions
Source: Insects. 2026 Sep 18;17(9):964. doi: 10.3390/insects17090964 (PMC13607452; doi:10.3390/insects17090964)
Supplement: Supplementary file 1 [file insects-17-00964-s001.zip › insects-4463411-Supplementary.pdf]

Supplementary Table S1. Published records used to construct the geographic distribution map of *Tropilaelaps* spp. infestations in *Apis mellifera* colonies.

Country/Region	First year shown on map*	References used
Philippines	1961	Baker & Delfinado (1961); Anderson & Morgan (2007)
India	1968	Crane (1968); Padhi & Rath (2012)
Vietnam	1968	Crane (1968); Anderson & Morgan (2007)
Afghanistan	1984	Woyke (1984)
Nepal	1985	Delfinado-Baker et al. (1985)
Papua New Guinea	1987	Delfinado-Baker (1987)**
Indonesia	1993	Matheson (1993); Gratzer et al. (2019)
China	1994	Woyke (1994); Luo et al. (2011)
Republic of Korea	2014	Jung et al. (2014)
Uzbekistan	2022***	Joharchi et al. (2025); Mohamadzade et al. (2024)
Russia	2021***	Brandorf et al. (2023, 2025)
Georgia	2024	Janashia et al. (2024)
Republic of Kazakhstan	2024***	Nuralieva et al. (2025)
Republic of Abkhazia (Gagra)	2025***	Brandorf et al. (2025)

* Year shown on the map corresponds to the first reported detection in *Apis mellifera* colonies, whereas the cited publication may have appeared later.

** The citation in your draft ("Delfinado-Bake, 1987") appears to contain a typographical error. The correct bibliographic information should be verified before submission.

*** The year shown on the map should correspond to the **first detection year**, not necessarily the publication year. For example, *Nuralieva et al.* (2025) reports the 2024 detection in Kazakhstan, and *Brandorf et al.* (2023, 2025) document detections that occurred earlier than publication.

Supplementary Table S2. Honey bee virus sequences retrieved from the NCBI GenBank database and used for primer design and sequence comparison.

**Virus**	**No.**	**Sequence name**	**NCBI accession No.**
Chronic bee paralysis virus (CBPV)	1	Chronic bee paralysis virus RNA 1, complete genome	NC_010711
	2	Chronic bee paralysis virus isolate CBPV-HB segment RNA1, complete sequence/ Chronic bee paralysis virus RNA 2, complete genome	NC_010712
	3	Chronic bee paralysis virus isolate CBPV-HB segment RNA2, complete sequence	MF175174
Deformed wing virus (DWV)	1	Deformed wing virus, complete genome	NC_004830
	2	Deformed wing virus capsid protein (gp1) gene, partial CDS	OQ819161
	3	Deformed wing virus isolate Tr_DWV_123-2 capsid protein (gp1) gene, partial CDS	OP504102
	4	Deformed wing virus isolate Tr_DWV_100-2 capsid protein (gp1) gene, partial CDS	OP504101
	5	Deformed wing virus type B isolate M55 polyprotein gene, partial CDS	MT675121
	6	Deformed wing virus type B isolate M53 polyprotein gene, partial CDS	MT675120
	7	Deformed wing virus type B isolate W11 polyprotein gene, partial CDS	MT675119
	8	Deformed wing virus type B isolate W75 polyprotein gene, partial CDS	MT675118
Israeli acute paralysis virus (IAPV)	1	Israel acute paralysis virus of bees strain DVE31-OP3-PA-USA-2007, complete genome	EU436423
	2	Israel acute paralysis virus of bees from USA, complete genome	EU224280
	3	Israeli acute paralysis virus isolate BJ2, complete genome	MG599488
	4	Israeli acute paralysis virus isolate IAPV-LN, complete genome	MZ269472
	5	Israeli acute paralysis virus strain PP, complete genome	KY243933
	6	Israeli acute paralysis virus strain Korea2, complete genome	KC690269
Kashmir bee virus (KBV)	1	Kashmir bee virus isolate KBV_NM RNA-dependent RNA polymerase and structural polyprotein genes, partial CDS	KF956375
	2	Kashmir bee virus isolate KBV_YC RNA-dependent RNA polymerase and structural polyprotein genes, partial CDS	KF956374
	3	Kashmir bee virus isolate KBV_GJ RNA-dependent RNA polymerase and structural polyprotein genes, partial CDS	KF956373
	4	Kashmir bee virus isolate KBV_NW RNA-dependent RNA polymerase and structural polyprotein genes, partial CDS	KF956372

Virus	No.	Sequence name	NCBI accession No.
	5	Kashmir bee virus isolate KBV_JC RNA-dependent RNA polymerase and structural polyprotein genes, partial CDS	KF956371
	6	Kashmir bee virus isolate KBV_GN RNA-dependent RNA polymerase and structural polyprotein genes, partial CDS	KF956370
	7	Kashmir bee virus isolate KBV_HD RNA-dependent RNA polymerase and structural polyprotein genes, partial CDS	KF956369
	8	Kashmir bee virus isolate KBV_AY RNA-dependent RNA polymerase and structural polyprotein genes, partial CDS	KF956368
Sacbrood virus (SBV)	1	Sacbrood virus isolate SBV/PNG-2014, complete genome	MT482477
	2	Sacbrood virus isolate MD1, complete genome	MG545286
	3	Sacbrood virus isolate API TEC MEX SBV2, complete genome	PP216851
	4	Sacbrood virus isolate API MONT MEX SBV1, complete genome	PP216850
	5	Sacbrood virus isolate SBV_MS, complete genome	MH267698
	6	Sacbrood virus strain RB Israel 2018, complete genome	OQ994751
	7	Sacbrood virus strain CAS1 polyprotein gene, complete CDS	PV239932
	8	Sacbrood virus strain Andrena RB Israel 2018, complete genome	OQ994752

Supplementary Table S3. Morphometric characteristics of adult female *Tropilaelaps mercedesae* from three geographic populations. Values are presented as mean ± standard deviation (SD). Model R^2, F statistics, and associated P values were obtained from one-way analysis of variance (ANOVA). Different superscript letters within rows indicate significant differences among populations according to Tukey's honestly significant difference (HSD) test ($P < 0.05$).

Character	Mean ± SD (Makhachkala) (n=29)	Mean ± SD (Zhetisay) (n=23)	Mean ± SD (Sochi) (n=23)	R^2	F	P
Body length (mm)	0.979 ± 0.059^{b}	1.004 ± 0.024^{a}	0.944 ± 0.034^{c}	0.243	95.84	<0.0001
Body width (mm)	0.536 ± 0.050^{a}	0.544 ± 0.038^{a}	0.484 ± 0.021^{b}	0.318	139.53	<0.0001
Abdominal shield length (mm)	0.525 ± 0.072^{b}	0.582 ± 0.045^{a}	0.515 ± 0.030^{b}	0.236	92.49	<0.0001
Abdominal shield width (mm)	0.105 ± 0.016^{a}	0.098 ± 0.010^{b}	0.098 ± 0.013^{b}	0.065	20.81	<0.0001
Anal shield A length (mm)	0.196 ± 0.021^{a}	0.189 ± 0.019^{b}	0.198 ± 0.022^{a}	0.037	11.50	<0.0001
Anal shield A width (mm)	0.128 ± 0.017^{b}	0.126 ± 0.015^{b}	0.134 ± 0.013^{a}	0.051	15.97	<0.0001

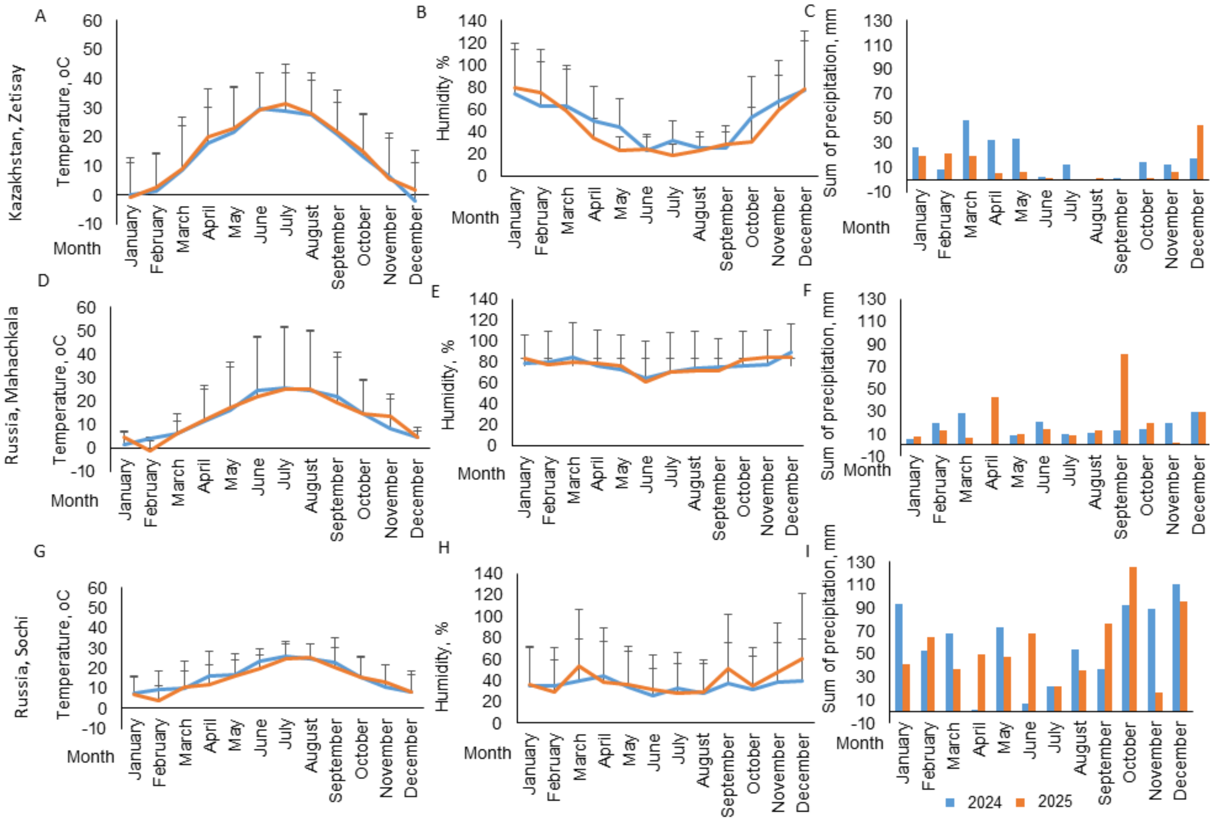

Supplementary Figure S1. Interannual comparison of monthly climatic conditions in Kazakhstan (Zetisay), Russia (Makhachkala and Sochi), between 2024 and 2025. Panels A–C represent Kazakhstan, D–F represent Dagestan, and G–I represent Sochi. Temperature panels (A, D, G) show monthly average temperatures with estimates of variability. Humidity panels (B, E, H) show the monthly average relative humidity. Precipitation panels (C, F, I) show monthly precipitation totals. Significant interannual differences were detected for temperature in Kazakhstan (Wilcoxon signed-rank test, $p = 0.039$) and humidity in Russia (Sochi) ($p = 0.040$), whereas no significant differences were observed for the remaining climatic variables (all $p > 0.05$). Russia (Makhachkala) exhibited the greatest climatic stability, with no significant interannual differences detected for temperature, humidity, or precipitation.

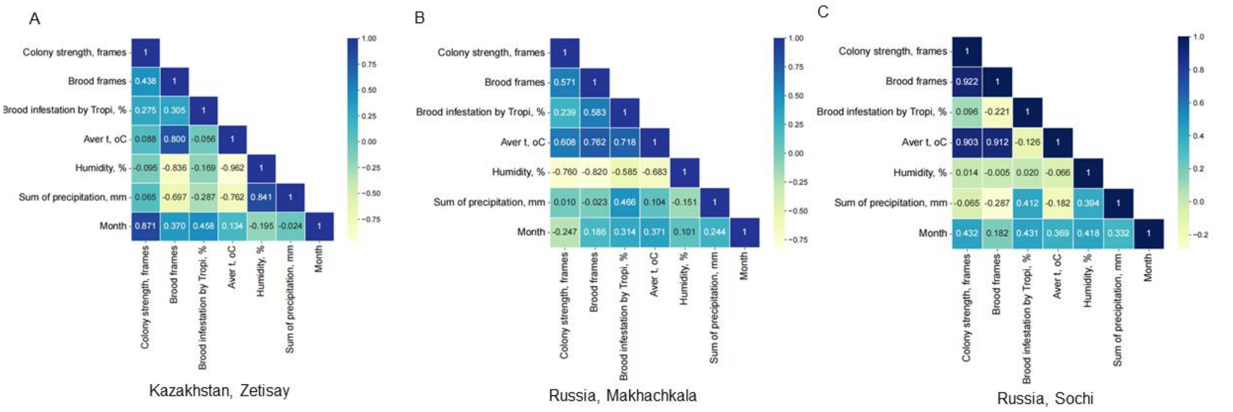

Supplementary Figure S2. Pearson correlation matrices for honey bee colony parameters, *Tropilaelaps* infestation, and climatic variables in Kazakhstan (A), Russia—Makhachkala (B), and Russia—Sochi (C) during 2025. Variables include colony strength (frames covered with bees), brood abundance (frames), brood infestation by *Tropilaelaps* mites (%), average temperature (℃), relative humidity (%), monthly precipitation (mm), and month. Correlation coefficients (r) are shown within cells, with color intensity indicating the strength and direction of the relationship.

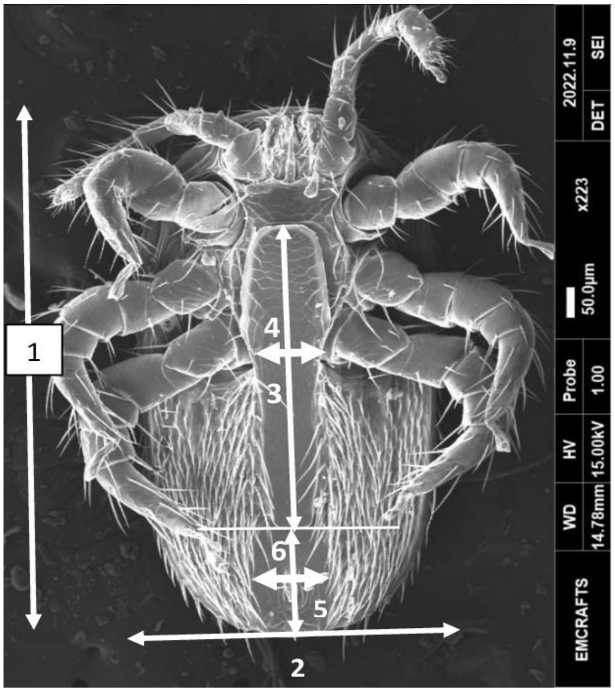

Supplementary Figure S3. **Morphometric measurements of *Tropilaelaps mercedesae*.** (1) dorsal shield length; (2) dorsal shield width; (3) epigynial plate length; (4) epigynial plate width; (5) ventrianal plate length; and (6) ventrianal plate width. Photograph by A.Z. Brandorf and A.S. Berezina (2022).
